# Antifungals susceptibility pattern of *Candida spp.* isolated from female genital tract at the Yaoundé Bethesda Hospital in Cameroon

**DOI:** 10.11604/pamj.2017.28.294.11200

**Published:** 2017-12-06

**Authors:** Michel Kengne, Siri Vivian Shu, Julius Mbekem Nwobegahay, Olivia Achonduh

**Affiliations:** 1 Medical Microbiology and Immunology Department, School of Health Sciences, Catholic University of Central Africa, Yaoundé, Cameroon; 2 Bethesda Hospital, Yaoundé, Cameroon; 3 Yaoundé Military Hospital, Cameroon; 4 Biotechnology Center, University of Yaoundé I, Cameroon

**Keywords:** Vaginal candidiasis, Candida spp, antifungals susceptibility

## Abstract

**Introduction:**

Vaginal candidiasis is considered as an important public health problem worldwide and its incidence has increased nowadays. In recent years, inappropriate and disproportionate use of antifungal drugs, automedication as well as non compliance have caused drug resistance.

**Methods:**

This study aimed at determining the *in vitro* antifungal susceptibility patterns of *Candida species*isolated from female genital tract at *Yaoundé Bethesda Hospital* in Cameroon. Two hundred and fourthy five women (age range: 15 years to 49 years) attending the hospital were recruited between January and June 2014 in this cross sectional study. Vaginal smears were collected using sterile swabs from each participant and cultured on sabouraud dextrose agar supplemented with chloramphenico l 0.5%; identification of *Candida spp.* was performed following standard methods. The disk diffusion method was used for antifungal susceptibility testing.

**Results:**

Out of the 245 vaginal smears collected, 94 (38.4%) strains of yeast were isolates among which 43 (45.7%) were *Candida albicans* and 51 (54.3%) were non albicans. The highest susceptibility of the isolates was seen for nystatin 62 (83.78%), ketoconazole 61 (82.43%) and fluconazole 60 (81.08%).

**Conclusion:**

Despite the noticeable resistance of *Candida spp.* isolates to miconazole and itraconazole, the results indicate that nystatin, ketoconazole and fluconazole are the drugs of choice for the therapy of vaginal candidiasis in this region.

## Introduction

Vaginal candidiasis (VC) is the most common opportunistic fungal infection that affects millions of women worldwide every year. It is caused by *Candida species* with *Candida albican* being responsible for the majority (80%-90%) of the cases [1]. VC is presently the first cause of vulvovaginitis in Europe and the second greatest cause in the United States and Brazil, where it is exceeded only by bacterial vaginosis [2]. Approximately 75% of adult women have at least one episode of vulvovaginal candidiasis in their lifetimes and about half of these women experience more than one recurrence; 5%-8% have multiple episodes each year [2]. VC usually occurs when there is an overgrowth of *Candida* present in the vagina as a normal commensal [3]. Pregnancy, contraception drugs with high estrogen, antibiotic consumptions, uncontrolled diabetes, immunosuppressive drugs, unsafe or excessive sexual intercourse, chronic anemia and season allergy are some predisposing factors of VC. The most common signs of VC are vaginal itching, dysuria and malodorous-white vaginal secretions [4]. Treatment of VC varies substantially and the most common drugs used are azole agents [5]. However, the widespread use of these drugs as prophylactic and therapeutic agents has been associated with the selection of less susceptible Candida strains, thereby causing serious problems in successful treatment of vaginitis [6]. Thus, early identification of *Candida species* and attention to their antifungal susceptibility patterns is very important for establishing strategies to control and/or prevent candidiasis by novel therapeutic management. This study aimed at determining the *in vitro* antifungal susceptibility patterns of *Candida species* isolated from female genital tract at *Yaoundé Bethesda Hospital* in Cameroon.

## Methods

### Sampling and culture

It was a prospective, descriptive cross-sectional study carried out at the Bethesda Hospital. The sampling technique used was a non probabilistic method of convenience. Participants for the study were recruited between January and June 2014 and were made of women attending the hospital for a medical follow up and complaining of various genital symptoms. Women who had a douche on the day of specimen collection, who were under any form of antifungal therapy and who were not at least 2 days away from the menstruation prior to specimen collection were not enrolled in the study. The sample size was calculated using the standard formula for sample size calculation (Lorentz’s formula):

$$N=\frac{z^{2}pq}{d^{2}}$$

Where z = the standard normal deviation at 1.96 (which corresponds to a 95 % confidence interval), p = the prevalence of candidiasis in Cameroon estimated at 35.4% [7]; q = 1-p and d = the degree of precision expected = 0.05. Based on these, our minimum sample size was 343 patients. Vaginal discharges were collected using sterile swabs from each participant and transported to the laboratory of the Yaoundé Central Hospital where they were cultured on sabouraud dextrose agar supplemented with 0.5% chloramphenicol. Plates were incubated at 35°C for 48h. Differentiation between *C. albicans* and non albicans isolates was done using the germ tube test and growth on chromogenic agar (gelose chromIDTM Candida, Biomerieux, France). Permission to conduct the study was obtained from the Yaoundé Bethesda and Central Hospitals. Informed consent was obtained from all study participants before they were enrolled.

**Antifungal susceptibility test:** The agar disk diffusion method was performed on the basis of the Clinical and Laboratory Standards Institute guidelines M44-A2 protocol for the evaluation of *Candida species* susceptibility to common antifungals [8]. All *Candida species* were subcultured at 35°C onto Sabouraud dextrose agar to ensure purity and viability then, plates surface containing Mueller-Hinton agar supplemented with 2% glucose and 0.5 µg/ml of methylene blue were inoculated using a sterile cotton swab dipped in a cell suspension adjusted to the turbidity of 0.5 McFarland standard. Itraconazole (10µg), nystatin (100 units), fluconazole (100µg), miconazole (50µg) and ketoconazole (50µg) discs (Becton Dickinson, Sparks, MD, USA) were placed onto the surfaces of the plates. Media were incubated for 48h for *C. glabatra* and 24h for the other *Candida species* at 35°C. The anti *Candida* activity was evaluated by measuring the diameter of the inhibition zone (mm) around the discs and the results were recorded as susceptible (S), susceptible dose dependent (SDD) and resistant (R). Reference strain of *C. albicans* (ATCC 2091) was used as control.

**Data analysis:** Data were recorded and analyzed on SPSS version 11.0 (SPSS, Inc., Chicago, IL). Discrete variables were expressed as frequencies and percentages.

**Ethics:** Permission to conduct this study was obtained from the School of Health Sciences ethics review committee and the Yaoundé Bethesda Hospital. Informed consent was obtained from all participants before their enrollment in the study.

**Limitation of the study:** The sample size was 245 participants instead of 343 as calculated. There was a difference of 98 participants due to the fact that we did not have many participants during our study period.

## Results

**Figure 1:**
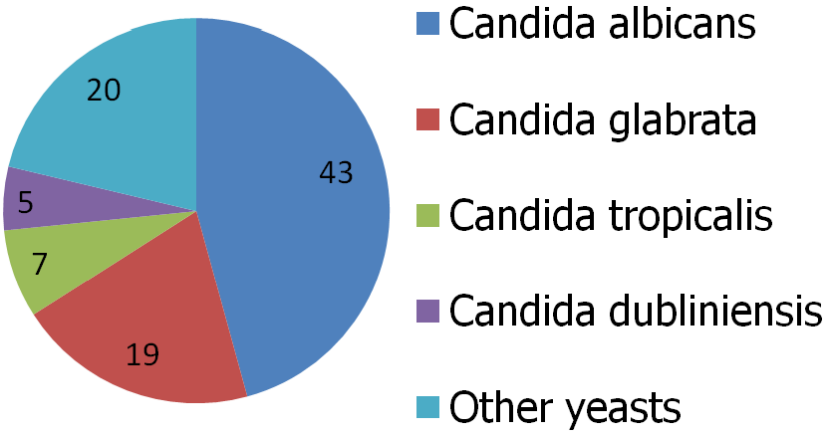
Frequency of isolated Candida species

Two hundred and forty five women, mean age 28.5 years (range 15-49) were enrolled in this study. Out of the 245 vaginal smears collected, 94 (38.4%) strains of yeast were isolates among which 43 (45.7%) were *Candida albicans* and 51 (54.3%) were non albicans (Figure 1). The overall *Candida species* recorded the highest susceptibility to nystatin 62 (83.7%), followed by ketoconazole 61 (82.4%), fluconazole 60 (81.1%), myconazole 44 (59.4%) and itraconazole 12 (16.2%). The highest resistance was seen for itraconazole 23 (31.1%) as shown in Table 1.

**Table 1:** Antifungals susceptibility pattern of the *Candida species* isolated

Antifungals tested		*Candida species* (no.)								Total	
		*C. albicans*(43)		*C. glabrata*(19)		*C. dubliniensis*(05)		*C. tropicalis*(07)			
		no	%	no	%	no	%	no	%	no	%
FLU	S	37	86.0%	16	84.2%	2	40.0%	5	71.4%	60	81.1
	SDD	3	9.1%	1	5.3%	0	0.0%	0	0.0%	04	5.4
	R	3	6.8%	2	10.5%	3	60.0%	2	28.6%	10	13.5
MCZ	S	27	61.4%	10	52.6%	3	60.0%	4	57.1%	44	59.4
	SDD	16	36.4%	9	47.4%	2	40.0%	2	28.6%	29	39.2
	R	0	0.0%	0	0.0%	0	0.0%	1	14.3%	01	1.3
ITR	S	8	18.2%	3	15.8%	0	0.0%	1	14.3%	12	16.2
	SDD	24	55.8%	10	52.6%	3	60.0%	2	28.6%	39	52.7
	R	11	25.0%	6	31.6%	2	40.0%	4	57.1%	23	31.1
KET	S	37	84.1%	15	78.9%	3	60.0%	6	85.7%	61	82.4
	SDD	6	13.9%	3	15.8%	2	40.0%	0	0.0%	11	14.8
	R	0	0.0%	1	5.3%	0	0.0%	1	14.3%	02	2.7
NY	S	38	86.4%	16	84.2%	3	60.0%	5	71.4%	62	83.7
	SDD	0	0.0%	0	0.0%	0	0.0%	0	0.0%	0	0
	R	5	11.6%	3	15.8%	2	40.0%	2	28.6%	12	16.2

FLU=fluconazole, MCZ=miconazole, ITR=itraconazole, KET= ketoconazole, NY=nystatin S= susceptible, I=intermediate, R= resistant

## Discussion

Researchers are interested in antifungal resistance because it is associated with elevated minimal inhibitory concentrations (MICs) that are associated with poorer clinical outcomes, breakthrough infections during treatment and increase healthcare costs. In this study, 43 isolates out of 94 isolates were *Candida albicans* and 51 (54.3%) were non albicans. These results are similar to previous studies [9] thereby showing the role of *C. albicans* in vaginal candidiasis where it had been established that its ability to form germ tube confers it survival abilities over other yeast species. Resistance of *Candida species* to the polyene and azole antifungals is the most prevalent type of resistance to antifungals; some of candidiasis therapy failures are due to the resistance of yeast pathogens to the drugs used. In the present study, the results of the susceptibility of the isolates to antifungals were as follows: fluconazole: 60 isolates (81.1%) susceptible and 10 (13.5%) resistant; miconazole: 44 isolates (59.4%) susceptible and 02 (2.7%) resistant; itraconazole: 12 (16.2%) susceptible and 23 (31.1%) resistant; ketoconazole 61 (82.4%) susceptible and 02 (2.7%) resistant; nystatin: 62 isolates (83.7%) susceptible and 13 (17.5%) resistant.

Our results further revealed that nystatin, fluconazole and ketoconazole were the most effective antifungal drugs and itraconazole had the poorest activity. Concerning *Candida* isolates susceptibility to nystatin (polyene), our results are in accordance with those of Jasem *et al.* [4]. Nevertheless, Ane-Anyangwe *et al.* [2] reported higher resistance (80%) of *Candida* isolates to nystatin which may be due to the excessive use of this drug as topical ointment or suppository as a result of its availability and low cost. As for *Candida* isolates susceptibility to fluconazole, the results of our study are supported by those of Pfaller *et al.* [10] who showed a high susceptibility to fluconazole of 90.2% out of 190,000 isolates from 41 countries; although evidence of resistance has been reported by some researchers [2-5]. VC is effectively treated with azole-based antifungal drugs [11] that may explain the resistance observed by these authors and the finding observed in our study with itraconazole.

## Conclusion

Our results show a variation in the array of the susceptibility of the *Candida species* isolated to the different antifungals tested with nystatin, ketoconazole and fluconazole being the drugs of choice for the therapy of vaginal candidiasis in this region. As a consequence, laboratory tests including species identification and antifungal susceptibility testing should be requested for women with vaginal candidiasis prior to drugs administration.

### What is known about this topic

- *Candida albicans* is the most important cause of vaginal candidiasis that affects women;
- Antifungal resistance is common and varies from countries and regions;
- Infections caused by resistant microorganisms often fail to respond to empiric treatment.

### What this study adds

- Information about the frequency of fungi species involved in vaginal candidiasis in the studied setting;
- Information about the antifungals to be used in treating vaginal candidiasis in the studied area.

## Competing interests

The authors declare no competing interests.
